# Estrogen Sulfotransferase Induction Inhibits Breast Cancer Cell Line MCF-7 Proliferation

**DOI:** 10.26717/bjstr.2019.22.003812

**Published:** 2019-11-13

**Authors:** Weiyu Jiang, Zhao Dai, Guangping Chen

**Affiliations:** 1 Department of Physiological Sciences, Center for Veterinary Health Sciences, USA; 2 School of Environmental and Chemical Engineering, China

**Keywords:** Estrogen Sulfotransferase, Estrogen-Dependent Breast Cancer, Flavonoid, Quercetin, Gene Regulation, Breast Cancer Treatment, Prevention

## Abstract

Estrogens are known for their proliferative effects, resulting in tumorigenesis and causing cancers. The majority of breast cancers are estrogen-dependent. Estrogenb-locking drugs developed for treating estrogen-dependent breast cancers include antagonists of estrogen receptors (ERs) and inhibitors of estrogen synthesis enzymes such as aromatase and steroid sulfatase (STS). However, drugs targeting estrogen inactivation enzyme, estrogen sulfotransferase (SULT1E1), have not been developed for estrogen-dependent cancer treatment or prevention. Estrogens must be inactivated after their actions *in vivo* by SULT1E1, uncontrolled estrogen activity in certain tissues will cause cancers. The majority of human breast cancer cell lines are known to express very low levels of SULT1E1 compared to normal mammary cells. Therefore, gene up-regulation of SULT1E1 could provide novel possibilities for the treatment of estrogen-dependent breast and endometrial cancers. Our data suggest that certain nutritional flavonoids induce SULT1E1 and inhibit cell proliferation in estrogen-dependent breast cancer MCF-7 cells. Our results also suggest that SULT1E1 inducer has an additive or synergistic effect on the inhibition of MCF-7 cell proliferation when combined with other known breast cancer drugs. Naturally occurring flavonoids are safe and inexpensive; they could be used for the prevention of certain breast cancers and/or used as co-drug for the treatment of estrogen-dependent cancers. These results may lead to creating innovative approaches for the treatment or prevention of estrogen-dependent cancers and may lead to the discovery of novel drugs or co-drugs.

## Introduction

Cytosolic sulfotransferases (SULTs) catalyze the sulfation of endogenous and xenobiotic hydroxyl-containing compounds [1]. Sulfation of xenobiotics is mainly associated with detoxification: sulfate esters are more water-soluble and more readily excreted by drug transporters than the parent compounds. SULTs are mostly studied as phase II drug-metabolizing enzymes. However, some SULTs show exclusive endogenous substrate specificity; sulfation is widely observed in various biological processes. The biological activities of various biological signaling molecules, including hormones (hydroxysteroids, thyroids, and glucocorticoids) and monoamine neurotransmitters, can be altered through sulfation. Sulfation normally leads to the inactivation of biological signaling molecules, as the sulfated forms are typically unable to bind to specific receptors. Sulfatases catalyze the de-sulfation [2]; together with SULTs, they regulate the functions and activities of various molecules.

Human estrogen sulfotransferase, SULT1E1, catalyzes the sulfation of estrogens in low nM concentration range. It is one of the key enzymes for the regulation and metabolism of estrogens [3]. Estrogens are known for their proliferative effects on estrogen sensitive tissues, resulting in tumorigenesis. Estrogens exist *in vivo* primarily as inactive sulfated forms [4]. SULT1E1 has been reported to play important roles in mammary cells for the regulation of estrogen activities [5]. Active free estrogens must be inactivated (sulfated) in estrogen-targeting tissues after their biological actions. Unregulated estrogen activities in estrogen-targeting tissues will lead to biological consequences. The majority of human breast cancer cell lines are known to express extreme low levels of SULT1E1 compared to normal human mammary epithelial cells [4]. Stable expression of SULT1E1 in human breast cancer cells has been reported to significantly inhibit cell growth at physiologically relevant estradiol concentrations [6]. It has been suggested that loss of SULT1E1 expression in the transformation of normal breast tissue to breast cancer is an important factor in increasing the growth responsiveness of preneoplastic or tumor cells to estrogen stimulation [6], and that factors involved in the stimulation of the SULT1E1 could provide new possibilities for the treatment of patients with hormone-dependent breast and endometrial cancers.

Naturally occurring flavonoids represent a major class of secondary metabolites in plants. They are widely distributed in plants including vegetables, fruits, and beverages such as tea, coffee, and wine [7]. Flavonoids are major functional components of many herbal drug preparations that have been used for thousands of years [8]. They are also major components of many modern dietary supplements [9]. These compounds are important health-enhancing food components and dietary supplements. Although they inhibit or kill many bacteria, they are much less toxic to humans and animals. Flavonoids interact with specific enzymes [10], bind to specific nuclear receptors [11], and scavenge free radicals [12]. In recent years, more and more reports have demonstrated that flavonoids bind to different kinds of nuclear receptors, including those that regulate drug-metabolizing enzymes, such as peroxisome proliferator-activated receptors (PPARs) [13], steroid hormone receptors (SHRs), and estrogen-related receptors (ERRs) [14]. The high binding affinity of flavonoids to various proteins is due to their special ring structures and multiple hydroxyl groups [15]. Our laboratory has investigated the regulation of human and rat SULTs by various flavonoids [16-19]. Our results demonstrate that some flavonoids strongly regulate hydroxysteroid SULTs; some flavonoids regulate SULT expression in reverse directions in hormone-producing tissues and hormone-targeting tissues [16]. Hydroxysteroid SULTs are much more sensitive to flavonoid regulation in hormone-producing and hormone-targeting tissues than in drug metabolizing tissues such as the liver in rats [16]. These results suggest that flavonoids may play important roles in the endocrine system by regulating SULTs.

In this work, we studied the gene regulation of human SULT1E1, in human breast cancer cell line MCF-7, by certain breast cancer drugs and flavonoids. We also studied the effect of cancer drugs and flavonoids on the cell proliferation of MCF-7 cells and investigated the combined effect of these drugs.

## Methods

### Materials

Adenosine 3’,5’-diphosphate disodium salt (PAPS), Eagle’s Minimum Essential Medium (EMEM) cell culture media, and 0.25% trypsin-EDTA were purchased from Sigma (St. Louis, MO). Fetal bovine serum used in the cell media was obtained from Hyclone (Logan, UT). SDS-polyacrylamide gel electrophoresis reagents and protein assay reagents were obtained from Bio-Rad (Hercules, CA). Western blot chemiluminescence reagent kits (Super Signal West Pico Luminol/Enhancer solutions) were purchased from Thermo Scientific (Rockford, IL). PVDF membranes used for Western blotting analyses were purchased from Millipore Corporation (Bedford, MA). [2, 4, 6, 7-^3^H (N)] Estradiol was ordered from PerkinElmer (Boston, MA). Rabbit anti-hSULT1E1 antibody [20,21] was a gift from Dr. David P Ringer (American Cancer Society). All other reagents and chemicals were of the highest analytical grade available.

### Cell Culture

MCF-7 cells (a human estrogen receptor-positive breast cancer cell line, ATCC number: HTB-22) obtained from American Type Culture Collection (Manassas, VA) were grown exactly as instructed by the supplier. 100 pg/ml estradiol was added to the medium for experiments as indicated in results section. Cells were maintained in a humidified atmosphere of 95% air and 5% CO_2_.

### Preparation of Cell Lysate

MCF-7 cells grown in control media or media containing 100pg/ml estradiol were washed twice with cold phosphate-buffered saline and incubated in lysis buffer (50 mM Tris-Cl [pH 7.5] containing 250 mM sucrose, 10 μg/mL trypsin inhibitor, and 10 μg/mL PMSF) for 15 minutes on ice. Cell lysates were cleared by centrifugation at 9000 g for 15 min, and the supernatant was used for Western blotting and enzyme activity assays, as previously described (Chen 2005, Maiti 2005).

### Enzyme Activity Assay

Estradiol sulfation activity by MCF-7 cell homogenate (9000 g supernatant) was determined by the radioactive assay method as described previously [22,23]. Briefly, sulfation activity was determined in a reaction mixture containing 50 mM Tris buffer, pH 6.2, 10 μM PAPS. [2, 4, 6, 7-^3^H (N)] Estradiol (diluted to 1.0 Ci/mmol; 0.15 μM final concentration) was used as substrate. 100 mg of MCF-7 cell homogenate was added to start the reaction. After 30 minutes incubation at 37°C in a water shaker bath, the reaction was stopped by adding 250 μl of 0.25 M Tris, pH 8.7. Extraction procedure was performed twice by adding 0.5 ml of water-saturated chloroform each time. After final extraction, 100 μl of aqueous phase was used for scintillation counting. The data shown in the Figure 3 are the average of three determinations of each time duplicated experiments.

### Western Blot Analysis of SULT1E1 Protein

MCF-7 cells were grown in 10 cm plates until reach 80% confluency. Then the cells were treated with drugs and cultured for 48 more hours. MCF-7 cell homogenate (9000 g supernatant) (30 μg) were subjected to Western blot analysis as described in our publications [24-26].

### MTT Assay

MCF-7 cells were grown in 96-well plates (100 ml/well) until reach 80% confluency. Then the cells were treated with drugs as described in results section and figure legends. After the treatments and 48 hours culturing, MTT is added to each well (final 0.5 mg/ml, stock solution 50 mg/ml in ehtanol) and incubated for 2 more hours at 37°C. After the incubation, medium is removed and the resulting formazan crystals are washed with PBS, then 200 ml of isopropanol is added to each well to dissolve the resulting formazan crystals. The plate is shaken on a rotating shaker for 5 minutes. Optical density is recorded at 540 nm using the POLARstar OPTIMA multi detection microplate reader (BMG LABTECH) in our laboratory. The cell proliferation of the control cells is considered as 100%, with treated cell proliferations expressed relative to the control. Six wells of isopropanol control reading average is subtracted for all experimental readings for each time of experiment.

### Statistical Analysis

For statistical analysis, an unpaired Student”s t-test was used for the comparison of values for a treated sample to that of the vehicle-treated sample to calculate the significance of difference. A two-way analysis of variance between groups (ANOVA) was performed to assess the significance of correlation for the inhibition of cell proliferation. Chou-Talalay method was used for the calculation of synergy for combined drug studies.

## Results

### Human SULT1E1 Gene Regulation by Breast Cancer Drugs in MCF-7 Cells

**Figure 1:**
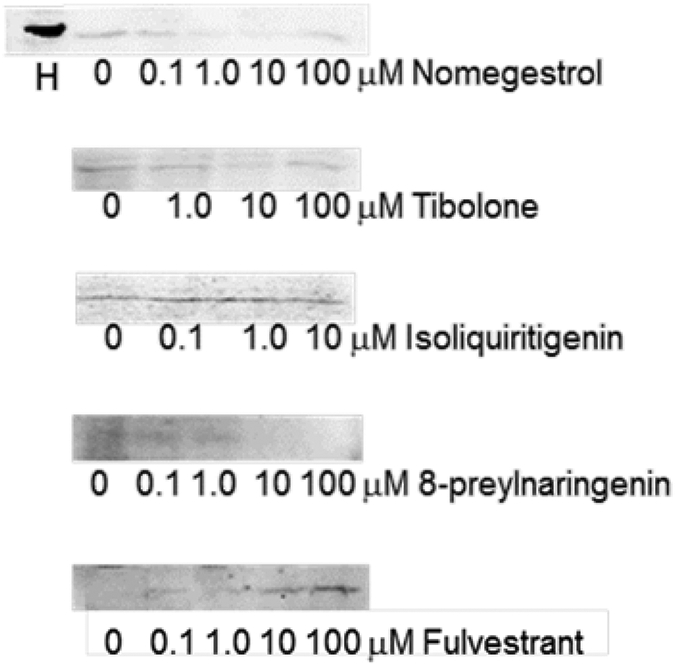
Breast cancer drug regulation of human SULT1E1 in MCF-7 cells. MCF-7 cells were treated for two days by drugs as indicated. 30 μ g of 9000 g supernatant of cell homogenates was used to run Western blot. Bands shown are closely under 34 kDa marker. H: 5 μ g of human intestinal cytosol was used as positive control. Rabbit anti-human SULT1E1 antibody from Dr. David Ringer was used for Western blot.

**Figure 2:**
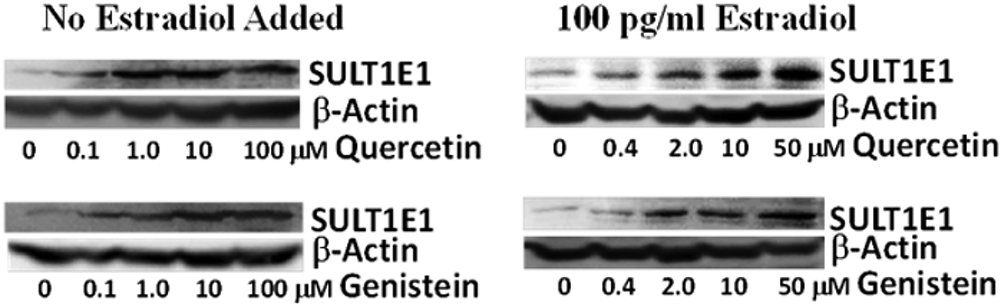
Flavonoid induction of human SULT1E1 in MCF-7 cells. MCF-7 cells were treated for two days by flavonoids at indicated concentrations. 30 μ g of 9,000 x g supernatant of cell homogenates were used to run Western blot. Rabbit anti-human SULT1E1 antibody from Dr. David Ringer was used for SULT1E1; monoclonal anti-β-actin–peroxidase antibody (Sigma, A3854) was used for β-actin.

Our SULT1E1 Western blot results indicate that control MCF-7 cells (30 mg of 9000 g supernatant of cell homogenates) showed a very weak band around 32 – 33 kDa (Figure 1 & 2). This is in agreement with literature reports that MCF-7 cells express extreme low levels of SULT1E1 [27]. Human intestine cytosol (5 mg) showed a relatively very strong band around 32 – 33 kDa (Figure 1). The extremely low level expression of SULT1E1 in MCF-7 cells caused the low quality pictures in Figure 1. For the breast cancer drugs studied, fulvestrant slightly induced SULT1E1 in a concentration-dependent manner, while other breast cancer drugs did not show significant effects on SULT1E1 expression in MCF-7 cells (Figure 1).

### Human SULT1E1 Gene Regulation by Flavonoids in MCF-7 Cells

We have studied flavonoid regulation of human SULT1E1 in MCF-7 cells. Our Western blot results suggest that some flavonoids significantly induce SULT1E1 in MCF-7 cells in the concentration range from 100 nM to 100 μM in a concentration-dependent manner. Figure 2 shows the results for quercetin and genistein induction of SULT1E1. These two flavonoids are more potent inducers than other flavonoids that we have studied. No estradiol was added for the results shown on the left, 100 pg/ml estradiol was added in the medium for the results shown on the right. Our results suggest that estradiol has no effect on flavonoid induction of SULT1E1. This agrees with reports that estrogens do not regulate SULT1E1 gene expression [28].

### Quercetin Induction of [3H] Estradiol Sulfation in MCF-7 Cells

**Figure 3:**
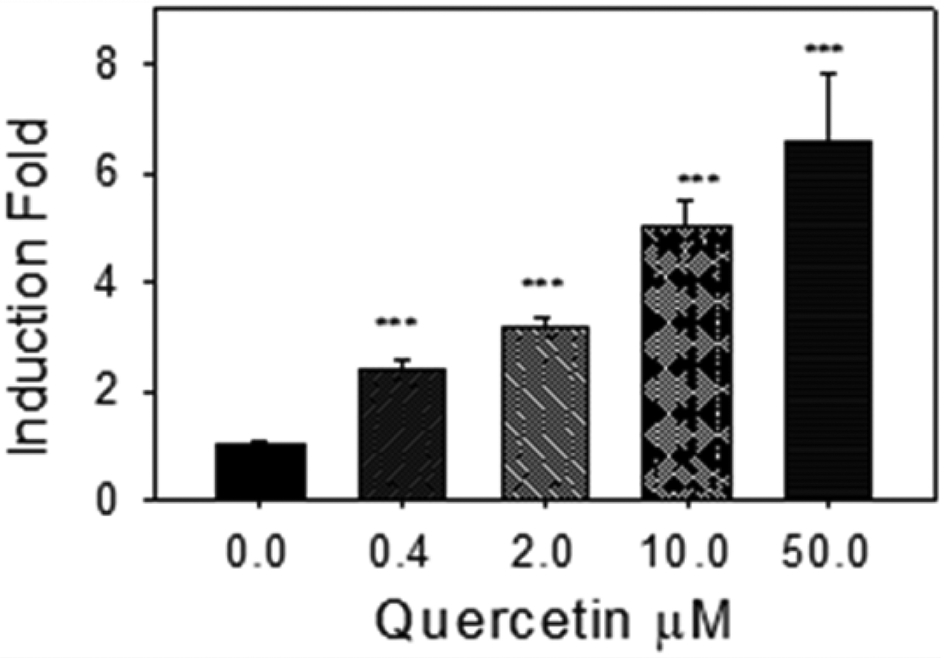
Quercetin induction of [3H] estradiol sulfation activity. 100 μ g of MCF-7 cell homogenate (9,000 x g supernatants) were used for enzyme activity assay. Control assay (0 μ M) specific activity was 0.21±0.02 pM/min/mg. ***: p<0.001

Our SULT1E1 enzyme activity assay ([3H] estradiol sulfation) results (Figure 3) agree with our Western blot results (Fig 2); quercetin significantly induced SULT1E1 enzyme activity in a concentration-dependent manner (Fig 3). 100 pg/ml estradiol was added to the MCF-7 cell culture medium for these experiments. Results in Figs 2 and 3 suggest that the induction of SULT1E1 protein by quercetin (Figure 2) increases the estrogen sulfation activity catalyzed by MCF-7 cell homogenate (9000 g supernatant) (Figure 3).

### Quercetin and Breast Cancer Drug Inhibiti on of MCF-7 Cell Proliferation

**Figure 4:**
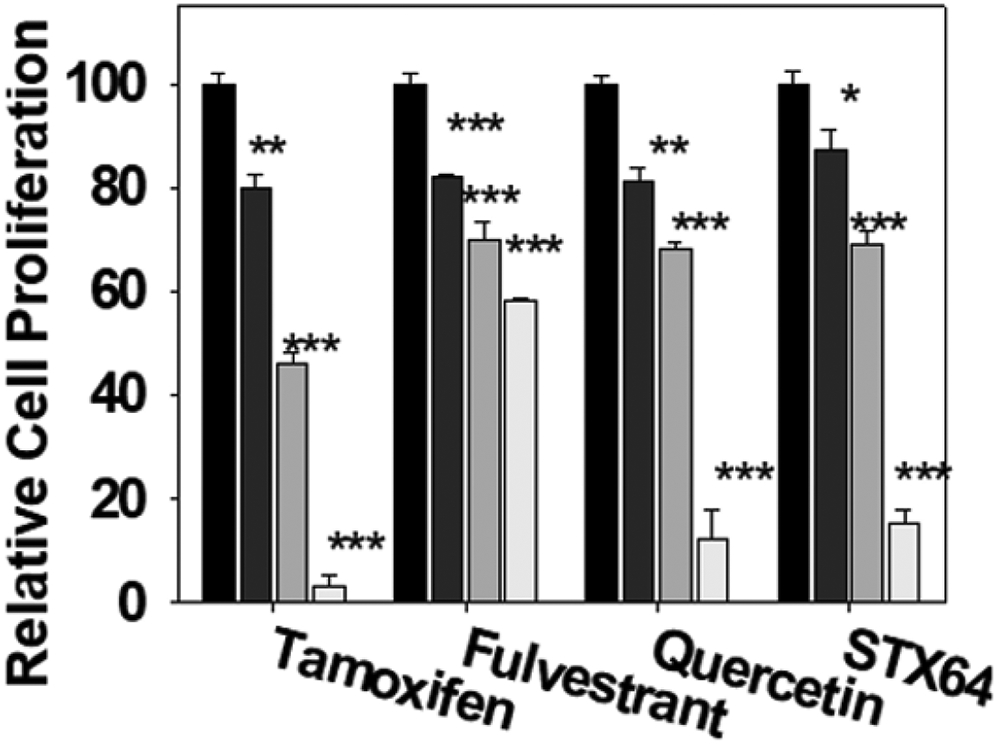
Inhibition of MCF-7 cells by quercetin and known breast cancer drugs. MCF-7 cells were treated with drugs at 0, 1.0, 10, and 100 μ M. for two days (medium containing 100 pg/ml estradiol). After the treatments, MTT 0.5 mg/ml (100 X stock solution in ethanol) was added to the medium and the cells were incubated for two more hours. Experiments were done in sextuplicate and were repeated three times. *: p<0.05; **: p<0.01; ***: p<0.001.

We have studied quercetin and known breast cancer drugs inhibition of MCF-7 cell proliferation using MTT assay. Our results show that quercetin and known breast cancer drugs (ER antagonists, tamoxifen and fulvestrant, and STS inhibitor STX64) significantly inhibit MCF-7 cell proliferation in a concentration-dependent manner (Figure 4). The cell proliferation inhibition potency of quercetin is less than that of the clinical drug tamoxifen, more than that of the clinical drug fulvestrant, and similar to the STS inhibitor STX64. The results suggest that quercetin (and other SULT1E1 inducers) has the potential to be developed as an anti-estrogen-dependent cancer drug. Our data also indicate that aromatase (estrogen synthetase) inhibitor anastrozole had no effect on MCF-7 cell proliferation (data not shown, 100 pg/ml estradiol in medium).

### Combined Effect of Quercetin, ER Antagonist, and STS Inhibitor on MCF-7 Cell Proliferation

**Figure 5:**
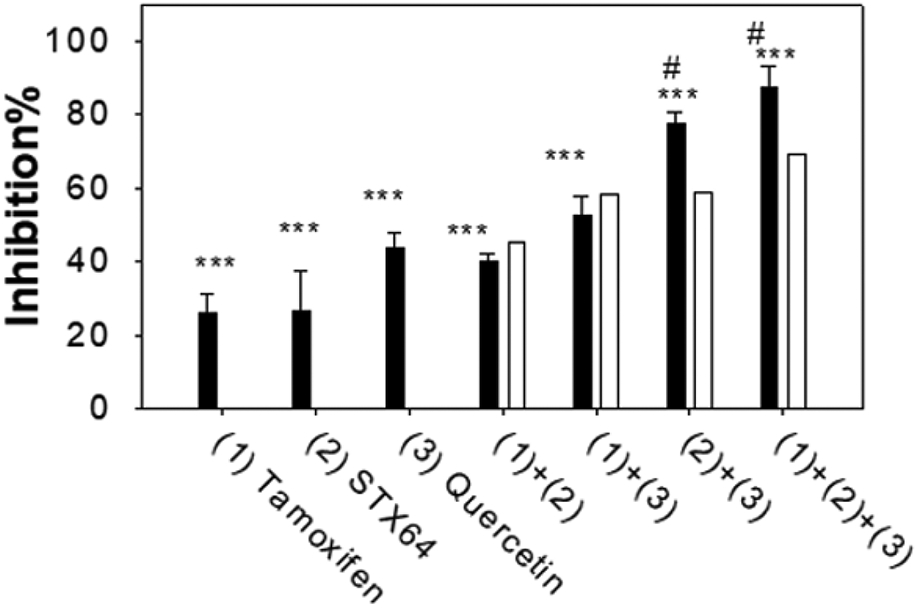
Combined effect of drugs on MCF-7 proliferation. Assays were done similar as described in Figure 4 Tamoxifen 5 μ M, STX64, 10 μ M, quercetin 30 μ M. Experiments were done in sextuplicate and were repeated 3 times. ***: p<0.001 for the cell proliferation inhibition significance compared to the vehicle treated cells. #: p<0.001 for the combined effect compared to the calculated simple additive effect.

We have studied the combined effect of SULT1E1 inducer (quercetin, 30 μM), ER antagonist (tamoxifen, 5 μM), and STS inhibitor (STX64, 10 μM) on MCF-7 cell proliferation using MTT assay (Figure 5). The filled bars in Fig 5 show the experimental results. The open bars show the calculated values suppose the combined drugs have only a simple additive effect. Two drugs synergistic effect is an effect greater than the sum (additive) of their individual effects. Our results suggest that when tamoxifen was combined either with STX64 ((1)+(2)) or with quercetin ((1)+(3)), the combined effect was basically additive, experimental value are not significantly different from the calculated additive value. When quercetin was combined with STS inhibitor STX64 ((2)+(3)) or with STX64 and tamoxifen ((1)+(2)+(3)), the combined effects were synergistic, experimental value are significantly higher than the calculated additive value (Figure 5). Quercetin induces SULT1E1 and increases the inactivation of estradiol to estradiol sulfate; while STX64 inhibits STS and decreases the activation of estradiol sulfate to estradiol. Our results suggest that the combined effect of SULT1E1 inducer and STS inhibitor is synergistic; the combined effect of ER antagonist with either SULT1E1 inducer or STS inhibitor has an additive effect.

## Discussion

Our laboratory has investigated flavonoid regulation of steroid SULTs in hormone-producing and hormone-targeting tissues [16-19] and the determination of the mechanisms driving the regulation [14,19,29]. Results reported here suggest that certain flavonoids strongly induce human SULT1E1 in MCF-7 cells (Figure 2). SULT1E1 inducer quercetin inhibits MCF-7 cell proliferation. Most importantly, our results suggest that SULT1E1 inducer show an additive effect with ER blocker and synergistic effect with STS inhibitor for the inhibition of MCF-7 cell proliferation. Our results suggest that SULT1E1 inducers can be used for the therapy of estrogen-dependent breast cancers or other estrogen-dependent cancers. These inducers may also be used for the prevention of breast cancers for women who have high risk for breast cancers.

Estrogens are known for their proliferative effects resulting in tumorigenesis in certain tissues. The majority of breast cancers are estrogen-dependent (or estrogen-sensitive). Current estrogen blocking drugs include antagonists of estrogen receptors and inhibitors of estrogen synthesis enzymes such as aromatase and steroid sulfatase. However, estrogen inactivation enzyme SULT1E1 has not been targeted to develop estrogen-dependent cancer drugs. There are no literature reports on studies of SULT1E1 gene induction in breast cancer cells. SULT1E1 is crucial for *in vivo* control of estrogen activities. Majority of human breast cancer cell lines express extreme low levels of SULT1E1 compared to normal mammary cells (Text Figure). Therefore, the up-regulation of SULT1E1 gene may provide novel possibilities for the treatment/prevention of patients with estrogen-dependent breast and endometrial cancers.


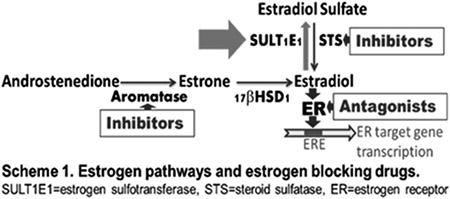


Naturally occurring flavonoids are bioactive food components. They are major functional components of many herbal drug preparations and many modern nutritional supplements. They are inexpensive and generally nontoxic. Our results suggest that some flavonoids can induce human SULT1E1 gene expression, and this increased SULT1E1 expression inhibits MCF-7 cell proliferation. Flavonoids could be used as co-drugs for estrogen-dependent cancer treatment. Our results provide essential information for future research, which may lead to creating innovative approaches for the treatment (and prevention) of estrogen-sensitive cancers and may lead to the discovery of novel drugs.
